# Hydrochlorate of Cocaine*Read before the Wayne County Medical Society, at the meeting held April 14, 1885, at Palmyra, N. Y.

**Published:** 1885-09

**Authors:** M. A. Veeder

**Affiliations:** Lyons, N.Y.


### * HYDROCHLORATE OF COCAINE.

M. A. VEEDER, M.D., LYONS, N.Y.

Although this drug has been prominently before the profession for a few months only, it has already been so thoroughly discussed in the medical journals that it is no longer to be regarded as a novelty. Nevertheless, the continued narration of experiences in regard to its properties and uses may not be unprofitable. The multiplication of testimony, based upon personal observation, will have the effect at least of developing greater exactness and thoroughness of knowledge. The following notes are to be taken for what they are worth, as a mere outline of the experiences of the writer in the use of the drug.

* Read before the Wayne County Medical Society, at the meeting held April 14, 1885, at Palmyra, N. Y.

In each case mentioned, unless otherwise specified, the strength of the solution employed was four per cent., as prepared by Dr. Squibb.

The first opportunity that presented itself for testing the capabilities of cocaine was in a case of disease of the rectum. There had been so much pain and tenesmus, that even digital examination was strenuously resisted by the patient. Ten drops of the four per cent. solution was carried beyond the sphincter ani muscle by means of a small syringe, and in a few minutes a satisfactory examination was made, both by means of the finger and the rectal speculum, the patient uttering no complaint whatever. The disease was found to be cancerous, which would account, perhaps, for the unusual sensitiveness of the parts when not benumbed by the anæsthetic.

In a case of laryngeal phthisis, the drug was given a thorough trial for the relief of the pain and spasm that attended every attempt to swallow even such articles of food as milk and broth. The remedy was applied to the throat by means of an ordinary medicine dropper, having a curved nozzle. The patient being requested to breathe through the mouth, at the moment when the palate was lifted, in the act of breathing, about five drops of the solution was projected downward, just back of the base of the tongue. In order to secure the full anæsthetic effect, it was found necessary that the throat should be as free as possible from mucus. Cocaine was thus used twice a day without harm, and with marked benefit so far as swallowing liquid food was concerned. The patient was made much more comfortable, not only whilst taking food, but, likewise, in the intervals, because of the increased quantity of nourishment received. Nor was there any marked increase of irritability, caused by the passage of the articles swallowed, across the diseased and tender surface. Occasionally, a little soreness was complained of an hour or so after a meal, but this soon disappeared and was regarded as unimportant. In fact, the general condition of the throat was, perhaps, slightly improved, although there was no permanent relief, it being found necessary to have the cocaine at hand for the relief of the pain experienced in clearing the throat of phlegm and mucus, as well as in swallowing, during the last few days of life.

It is, perhaps, in just those operations about the eye that fall within the province of the general practitioner, that cocaine will be found to be of the most real service. I have derived great satisfaction from the use of it for the purpose of aiding in the extraction of fine particles of metal that had become imbedded upon the globe of the eye; also, in picking out from the palpebral conjunctiva the little seed-like bodies that are the source of so much irritation in the disease known as miliary trachoma. I have not seen much benefit from its use in the operation of probing the lachrymal duct, but still there may be cases in which it will be found to be of great use. It certainly has the power of relaxing spasm in circular muscles in some instances at least.

In employing cocaine about the eye, there is a point that demands notice, because of its bearing upon the question as to possible poisonous effects of the drug. It should always be borne in mind that the anæsthesia produced by it differs from that to which we have otherwise become accustomed in being purely local. The patient is fully conscious as to what is being done, though he may feel no pain. Now, there are persons who faint during so painless an operation as vaccination, and it is not strange that there are those who are made to feel sick and faint when they see an instrument that looks increasingly formidable, as it comes straight down upon the eye whose gaze is fastened upon it. The sensations thus experienced may, without doubt, be justly ascribed to the nervous condition of the patient, rather than to any poisonous effect of cocaine—but more of this anon.

In using the laryngoscope, and in removing foreign bodies from the throat, cocaine renders valuable assistance. The cheesy nodules that form, as the result of follicular catarrh of the tonsils, are often difficult to remove, and cause much irritation

5

by their presence. The application of cocaine solution to the tonsil permits it to be scraped and manipulated by the curette, or some similar instrument, so that these bodies may be removed far more easily and completely than is otherwise possible.

Diluted with water, and thrown into the nostrils, the cocaine solution causes the mucous membrane, as far as visible, to become paler. Probably the congestion due to catarrh may be somewhat relieved in this way. It is probable that, as the drug becomes cheaper, it will be thoroughly tested in such conditions, and especially in hay fever.

As a remedy for earache, cocaine evidently has a future, although it may not always succeed so promptly and thoroughly as it did in a case that I saw recently. Applied to the swollen gums of children who are cutting teeth, it gives relief from pain, although its action, even upon mucous surfaces, is very superficial, so that it is not of much service in cutting operations generally. In dentistry, its chief use will probably be as an application to cavities in teeth that are in process of being cleaned and scraped. In the case of cavities in aching teeth, which are sometimes brought to the doctor for temporary relief, my routine is as follows: I apply to the cavity three pledgets of cotton, one after another, the first moistened with cocaine solution, the second with strong carbolic acid, and the third with glycerine. The first benumbs, the second cauterizes, and the third prevents undue action of the acid. The patient is dismissed with the injunction not to attempt to bite anything with the tooth that contains the cavity.

An injection of ten drops of cocaine solution, diluted with thirty drops of water, relieves chordee very promptly. The unhappy sufferers from chancroidal and other ulcers are afforded much consolation by the application of cocaine to their sores, preparatory to the much-dreaded "burning" that they are pretty apt to receive at the hands of the faithful practitioner.

Rubbed upon the unbroken skin, cocaine in the ordinary form of solution has little, if any, effect. I have tried it in supra-orbital neuralgia; thus far, have not met much occasion for encouragement to persist in its use in this way. Perhaps the oleate of cocaine, like the oleates of morphia and aconitia, may prove effectual when rubbed upon the skin. Where the skin is broken, as in bunions and frost-bites, the cocaine solution proves to be very efficient. I have not had occasion to use it upon very large surfaces that have thus been denuded. It is not likely, however, that it would have any injurious effect, inasmuch as its action appears to be almost purely local. Injecting it under the skin by means of the hypodermic syringe, only slightly extends the area and depth of the anæsthesia produced by it. Moreover, the following case, published in a German medical periodical as long ago as 1863, and quoted in the *Fractitioner*, would appear to prove conclusively that cocaine is not actively poisonous, even when taken into the stomach in immense doses.

"An apothecary intending to commit suicide extracted twenty-four grammes of crystallized substance from two pounds of coca leaves, and administered it to himself in a glass of beer, afterwards taking two small glasses of brandy. For a time he felt quite well and soon went to sleep, but woke up with gripings of the stomach, burning pains in the palate, dryness of the throat and mouth, dizziness, great weakness of the whole body, perfect consciousness, pulse and temperature normal. After taking 1¼ grains of morphia, he fell asleep and woke up quite well, though he did not pass urine for twenty-four hours."

It has not been my purpose to attempt to give a summary of the history of the drug and its physiological effects, which may be found, fully elaborated, in almost all recent medical publications. I shall have accomplished all that I intended if, by mentioning these bits of practical experience, I have been able to enlighten, in the least, any in regard to the capabilities of a drug which, evidently, has a multitude of uses and few disadvantages.

